# Chronic mechanical irritation as a potential contributor to nasal fibroma formation in a captive Javanese cownose ray (*Rhinoptera javanica*)

**DOI:** 10.3389/fvets.2026.1874543

**Published:** 2026-08-12

**Authors:** Ji-Hyung Park, Kyungwhan An, Sungwon Jeon, Jong Bhak, Seung Hyeok Seok, Sang Wha Kim

**Affiliations:** 1 College of Veterinary Medicine & Institute of Veterinary Science, Kangwon National University, Chuncheon, Gangwon, Republic of Korea; 2 Korean Genomics Center (KOGIC), Ulsan National Institute of Science and Technology (UNIST), Ulsan, Republic of Korea; 3 AgingLab, Inc., UNIST, Ulsan, Republic of Korea; 4 Macrophage Lab, Department of Microbiology and Immunology, Institute of Endemic Disease, Seoul National University College of Medicine, Seoul, Republic of Korea; 5 Department of Biomedical Sciences, Seoul National University College of Medicine, Seoul, Republic of Korea; 6 Cancer Research Institute, Seoul National University, Seoul, Republic of Korea; 7 Institute of Endemic Diseases, Seoul National University Medical Research Center, Seoul, Republic of Korea

**Keywords:** artificial environment, captive breeding, Chondrichthyes, *de novo* assembly, fibroma, nasal tumor

## Abstract

**Introduction:**

Rays rarely develop neoplasia in nature, yet tumors have been repeatedly reported in captive individuals, suggesting environmental or species-specific factors.

**Methods:**

We surgically removed the nasal fibroma under general anesthesia, followed by transcriptomic and histopathological analyses in a captive Javanese cownose ray (*Rhinoptera javanica*).

**Results:**

Histopathology revealed a fibromatous lesion characterized by dense, mature collagen-rich fibrosis in the central core, accompanied by peripheral fibroinflammatory change, vascular congestion, hemorrhage, and inflammatory-cell infiltration. Exploratory transcriptomic profiling of the central fibrotic core provided complementary evidence of candidate remodeling-associated expression patterns, including increased mechanical-stimulus response and epithelial proliferation terms. Together, these findings support a plausible link between repeated surface contact and chronic injury-associated remodeling of the nasal tissue.

**Discussion and conclusion:**

These findings suggest that persistent mechanical stimulation in artificial environments may contribute to chronic fibrosis and fibroinflammatory tumor development. Together with the clinicopathological findings, the transcriptomic findings provide exploratory, hypothesis-generating support for a possible association between captivity-related mechanical stress and species-specific fibroma formation in the Javanese cownose ray.

## 1 Introduction

Aquariums serve as valuable observational windows for marine biologists, offering opportunities to study marine lives under controlled conditions. Animals housed in aquariums are under constant surveillance, which facilitates the early detection and investigation of disease conditions, often more readily than in wild populations. However, such advantages are counterbalanced by the inevitable differences between natural habitats and artificially constructed, nature-mimicking aquatic environments. These artificial systems often impose unique physical, chemical, and biological stimuli on the animals due to their confined space, unnatural water flow and quality, the presence of artificial wall and floor structures, and high stocking densities. The abrupt transition from evolutionarily adapted wild habitats to artificial settings may lead to the emergence of disease conditions that are not evolutionarily anticipated.

Mechanical stress arising from artificial environments is a well-recognized pathogenic factor known to drive chronic wound repair and fibrotic remodeling across diverse species (1). Persistent mechanical stimulation can provoke inflammatory responses within tissues, leading to fibroblast activation and excessive collagen deposition, ultimately resulting in aberrant wound healing processes (2). These mechanisms are well-documented in various terrestrial animal models, including mammals (3), and have recently been reported in piscine species such as zebrafish (4).

Chronic mechanical stress not only promotes fibrotic responses but also lays the pathological groundwork for abnormal tissue proliferation and tumor development. For instance, pulmonary fibrosis is associated with an increased risk of pulmonary adenocarcinoma (5), and liver fibrosis resulting from chronic hepatic injury is a known promoter of hepatocellular carcinoma (6). Similar inflammation-fibrosis-tumorigenesis cascades have been demonstrated in aquatic models, including zebrafish (7), underscoring the potential of physical stimuli as a tumor-promoting factor in fish species.

The Javanese cownose ray (*Rhinoptera javanica*, also known as the flapnose ray) belongs to the order Myliobatiformes within the subclass Elasmobranchii and is characterized by a broad cephalic lobe. This species inhabits coastal tropical and subtropical waters and is currently listed as “Endangered” on the IUCN Red List (8). Despite its conservation status, the Javanese cownose ray is widely maintained in large aquariums for educational and exhibition purposes. It is commonly found in major public aquariums across Korea, Japan, and Southeast Asia, and is one of the more publicly recognizable elasmobranch species.

Elasmobranchs, including sharks and rays, have long been recognized for their low incidence of neoplasia (9, 10), and indeed, only a limited number of tumor cases have been documented to date. Reported neoplastic cases in rays have all involved captive-bred individuals in aquariums, with most studies focusing primarily on tumor occurrence and histopathological characterization (10–13). In contrast, no reports exist for wild populations, suggesting that tumor formation is rare in natural environments. This disparity indicates that tumors in artificial or captive settings are likely influenced by environmental factors such as spatial constraints, mechanical stress, and water quality conditions. Therefore, beyond descriptive pathology, it is crucial to investigate the causal and mechanistic pathways underlying tumor formation in these species. To achieve this, transcriptomic profiling should be integrated with traditional histopathological analyses to elucidate the molecular mechanisms of tumorigenesis in elasmobranchs (14).

In this study, we performed transcriptomic analysis of nasal fibroma in the Javanese cownose ray maintained in an artificial aquatic environment to investigate the etiology of the lesion. To our knowledge, this represents the first transcriptomic characterization of a tumor in an elasmobranch species. As no reference-quality genome currently exists for the Javanese cownose ray, we performed *de novo* transcriptome assembly and generated a foundational dataset. Gene expression profiles between tumor and matched normal tissues were used to identify enriched gene sets and infer functional significance via gene ontology analyses. By integrating transcriptomic data with histopathological features and environmental context, we aim to identify potential contributing factors associated with lesion development. Ultimately, this study provides insights into the pathological mechanisms and fibrotic tumorigenesis in Javanese cownose ray and highlights the link between chronic mechanical stress in artificial environments and disease manifestation in elasmobranchs.

## 2 Materials and methods

### 2.1 Animal and husbandry conditions

A female, 18.2 kg Javanese cownose ray was housed at a local aquarium in the Republic of Korea and had been under human care for nine years. The ray was kept in the main tank of the aquarium (2,000 tons of water; 10 m of depth). Water quality was maintained as follows: temperature: 24 ± 1 °C; pH: 7.6 ± 0.1; salinity: 32 ± 2 ppt, and the water filtration system was consisting of skimmers, sand filters, and ozone purifier. The ray was fed twice a day with a diet of squid, herring, mackerel, white leg shrimps, supplemented with multivitamins (Mazuri shark & ray, 5MD8).

Nodular lesions were first observed in both nostrils of the ray in 2020 and gradually grew up to become the sizes of 3.2 cm × 2.7 cm (left nostril mass; width × length) and 0.8 cm × 0.8 cm (right nostril mass) when first operative resection proceeded on February 15, 2022.

### 2.2 Operations and clinical procedures

#### 2.2.1 Primary operation

Prior to anesthesia, the Javanese cownose ray underwent fasting over 24 h. Anesthesia induction was performed using 100 ppm of neutral-buffered MS-222 (3-Aminobenzoic acid ethyl ester). Ten minutes after an excitatory stage, respiratory rate decreased and movement reduced. Subsequently, a combination of butorphanol (0.5 mg/kg) and medetomidine (0.05 mg/kg) was administered to induce deep general anesthesia (15). The ray was then transferred to the surgery table covered with wet towel and preoperative antibiotics (enrofloxacin 5 mg/kg) was injected.

To maintain anesthesia, seawater containing 50 ppm neutral-buffered MS-222 was continuously supplied to the gill through the oral cavity of the ray. Local infiltration anesthesia was achieved using 2% lidocaine around the tumor mass. Excision was performed with a narrow margin (<0.2 cm) to minimize surgical invasiveness. The skin was sutured using a simple interrupted technique with non-absorbable 4–0 Nylon suture.

After the surgery, the ray was housed in a recovery tank with a depth of 1.0 meters to stay isolated from other animals, and silver sulfadiazine (Silmazin 1% cream) was applied to the surgical site. Oral antibiotics (enrofloxacin 5 mg/kg, po, sid) were administered along with diet.

#### 2.2.2 Second operation

Four months after the first surgery, the sizes of the recurred masses had significantly increased (right side: 2.7 cm × 2.5 cm × 3.3 cm; left side: 3.1 cm × 3.2 cm × 3.0 cm; height × width × length) compared to the original masses. Anesthesia was induced with the same protocol used in the first operation. Wider margin excision was proceeded with over 0.5 cm surgical margin, and secondary wound healing was induced. Post-recovery management time in the recovery tank was shortened, and the ray was transferred to its original main tank as soon as possible.

### 2.3 Tissue sample collection for histopathology and transcriptomic analysis

Tissue samples for histopathologic and transcriptomic analyses were collected during the second operation. Nasal tumor masses were cross-sectioned, and half of the mass was fixed with neutral-buffered formalin (10%) for histopathological analysis.

**Figure 1:**
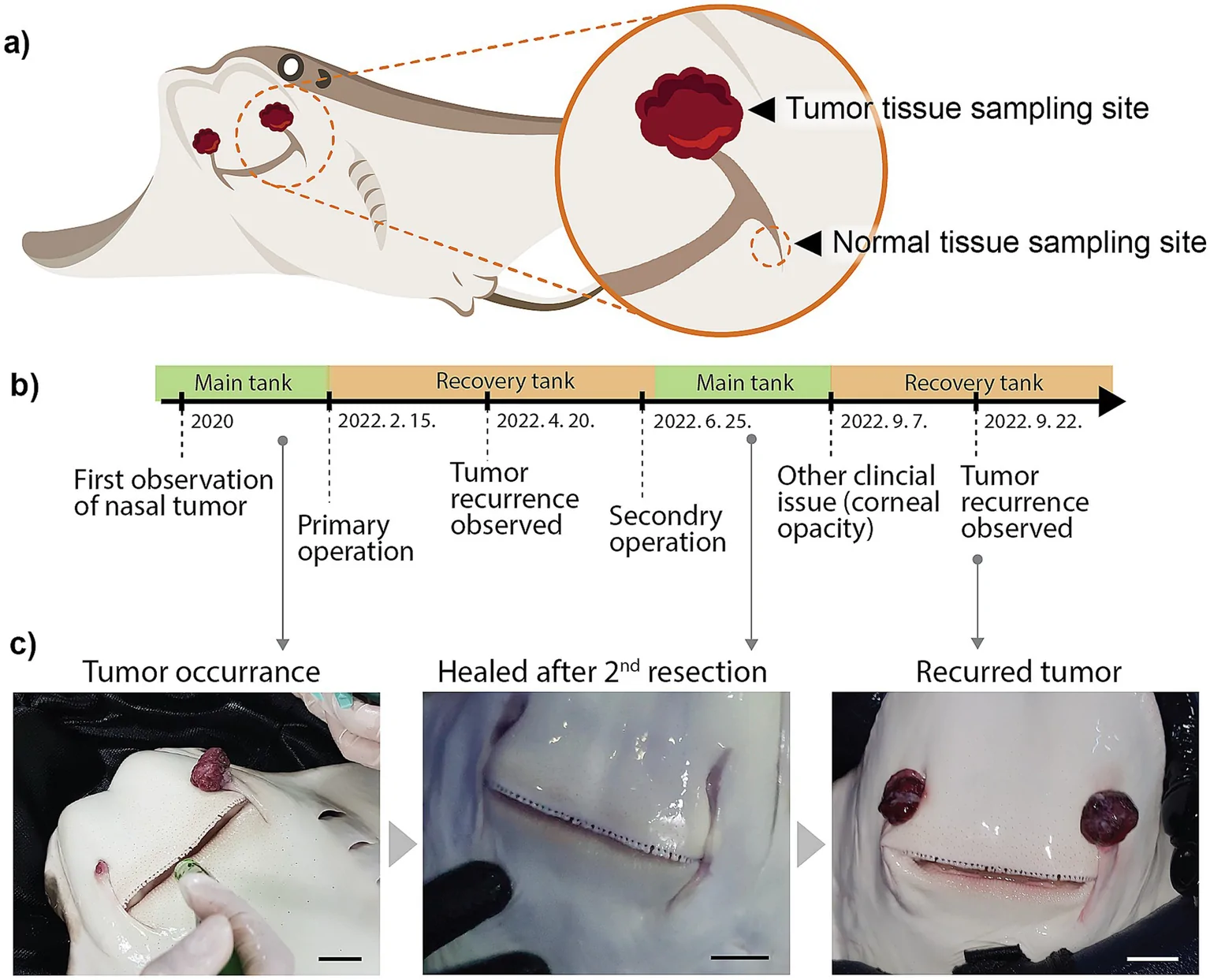
Anatomical location and clinical history of the nasal tumor in the Javanese cownose ray (*Rhinoptera javanica*) **(a)** Schematic illustration of the Javanese cownose ray at the time of the second operation, indicating the location of nasal tumors. Orange circles denote tissue sampling sites for transcriptomic analysis. **(b)** Timeline summarizing the clinical history, including initial tumor detection, surgical interventions, and recurrences, annotated with the corresponding housing conditions (main tank vs. recovery tank). **(c)** Serial photographs of the nostril region captured throughout the clinical history. Scale bars = 3 cm.

For transcriptomic analysis, tissue was sampled from the central portion of the left nasal tumor mass. This region was selected because it represented the main fibromatous component of the lesion and was therefore considered the most appropriate site for exploratory profiling of molecular features associated with fibrotic tumor tissue, including possible injury-associated programs. This sampling strategy was also to minimize contributions from the reactive peripheral regions, where histopathology showed more prominent fibro-inflammatory activity, hemorrhage, and inflammatory-cell infiltration. In addition, because the tumor was surgically excised and sampled grossly, central-core sampling was intended to reduce potential contamination from adjacent grossly normal tissue at the excision margin, where tumor tissue, reactive interface, and surrounding non-tumor tissue could be mixed. Matched normal tissue was collected from grossly normal perioral skin on the ipsilateral (left) side, away from the nasal mass (Figure 1a). This location was selected to avoid the grossly evident lesion while maintaining an anatomically comparable sampling site within the same facial region.

Tissue specimens intended for RNA analysis were placed into sterile cryogenic tubes, snap-frozen in liquid nitrogen, transported on dry ice, and maintained at −80 °C until RNA extraction and library preparation. Tumor and normal specimens were handled under the same preservation and storage conditions to minimize RNA degradation and reduce technical variation attributable to sample processing.

### 2.4 Histopathology analysis

Tissue samples were fixed in 10% neutral-buffered formalin for 24 h, followed by routine histological processing, including paraffin embedding, microtome sectioning, deparaffinization, staining, and slide mounting. Histological sections were stained with hematoxylin and eosin (H&E) for general tissue morphology and with Masson’s trichrome (MT) to assess collagen deposition and fibrotic changes.

### 2.5 RNA library construction and sequencing

Total RNA was isolated from cryopreserved tissue specimens using the MGIEasy RNA Prep Kit (MGI, #MG1000006386), following the manufacturer’s instructions. Prior to library construction, RNA quantity was measured using Qubit fluorometry, and RNA integrity was assessed on an Agilent 4,150 TapeStation system (Agilent Technologies, #G2992A). The matched normal tissue sample yielded 49.5 μg of total RNA with a RIN of 8.7. The tumor RNA sample obtained from the central portion of the left nasal tumor mass yielded 51.3 μg of total RNA with a RIN of 8.3. Both RNA samples passed the sequencing provider’s RNA sample quality-control assessment before library preparation (Supplementary Table S1).

Following RNA quality assessment, an aliquot of total RNA from each sample was subjected to poly(A) + selection using the Dynabeads^™^ mRNA Purification Kit (Thermo Fisher Scientific, #01152851), which captures polyadenylated transcripts via oligo(dT)-coated magnetic beads. The resulting mRNA was then used for directional library preparation with the MGIEasy RNA Directional Library Prep Set (MGI, #MG1000006386), enabling strand-specific transcriptome profiling.

Library fragment size distribution was assessed on an Agilent 4,150 TapeStation system (Agilent Technologies, #G2992A) using the High Sensitivity D1000 ScreenTape Assay (Agilent, #5067–5,582) to confirm appropriate insert sizes and the absence of primer-dimer artifacts. Final library concentrations were determined using the Qubit 2.0 Fluorometer (Thermo Fisher Scientific, #Q32866) in combination with the Qubit RNA High Sensitivity Assay Kit (Thermo Fisher Scientific, #Q32854). The normal tissue library had a peak fragment size of 301 bp, concentration of 21.4 ng/μl, and qualified yield of 3.2 pmol. The tumor library had a peak fragment size of 297 bp, concentration of 29.0 ng/μl, and qualified yield of 4.4 pmol. Both libraries passed the sequencing provider’s library quality-control assessment.

Paired-end RNA sequencing (2 × 150 bp) was conducted on the DNBSEQ-T7 platform (MGI), selected to provide deep, high-throughput coverage optimal for comprehensive *de novo* transcriptome assembly. We targeted sequencing depth to minimum 20 Gb per sample to maximize the detection of both abundant and rare transcripts, as well as to support accurate reconstruction of full-length isoforms.

### 2.6 Pre-assembly processing and quality control

Raw paired-end RNA-seq reads were preprocessed using fastp v0.23.1 and quality-assessed with FastQC v0.11.8 before and after filtering. Paired-end adapter sequences were detected automatically. Fixed end trimming removed the first 15 bases and final 5 bases from both read pairs, followed by sliding-window quality trimming at a mean threshold of Q20. Reads were retained if they contained no more than one ambiguous base and had a minimum average quality of Q20.

Preprocessing retained 156,866,896 of 159,017,210 reads from the tumor library (98.65%) and 207,832,450 of 210,879,874 reads from the normal tissue library (98.55%). Post-filtering Q30 rates increased from 93.13 to 94.22% in the tumor library and from 93.09 to 94.26% in the normal tissue library. Removed reads were predominantly too short or contained excess ambiguous bases, whereas only 6,062 tumor-library reads and 9,686 normal-library reads were eliminated because of low sequence quality (Supplementary Table S2).

### 2.7 *De novo* transcriptome assembly

High-depth RNA-seq read pairs (78,433,448 for tumor and 103,916,225 for matched control) were used for *de novo* transcriptome assembly of *Rhinoptera javacina*, a non-model organism with no reference genome. The assembly was conducted on a 2 TB RAM, 18-core high-performance computing server powered by Intel^®^ Xeon^®^ E7-8860 v4 @2.20GHz CPUs with 70 threads using Trinity (v2.14.0). *In silico* normalization was performed using Trinity’s built-in *insilico_read_normalization.pl* script to reduce redundancy and optimize memory usage during the assembly, leaving 20.65% of total reads (37,650,608). Required dependencies included Bowtie2 (v2.3.2), samtools (v1.12.0), Jellyfish (v2.3.0), and Salmon (v1.19.0).

### 2.8 Post-assembly quality control

Transcriptome assembly statistics, including total contig count, N50 values, and transcript length distribution, were computed using Trinity’s built-in Trinitystats.pl. script. To evaluate mapping efficiency, trimmed RNA-seq reads were aligned back to the assembled transcriptome using Bowtie2 (v2.3.2).

Read alignment statistics were examined to evaluate how effectively the RNA-seq reads were represented in the *de novo* transcriptome using samtools view (v1.3.0). Unique mapping was defined at the read-pair level as mapped read pairs without secondary alignment records, whereas multi-mapped read pairs were defined as those with one or more secondary alignments. For the normal tissue library, 80,572,107 (77.54%) mapped read pairs were recovered from 103,916,255 read pairs retained after quality control, of which 21,528,256 (20.72%) were uniquely mapped and 59,043,851(56.82%) were multi-mapped. For the tumor library, 62,613,361 (79.83%) mapped read pairs were recovered from 78,433,448 read pairs, of which 17,130,243 (21.84%) were uniquely mapped and 45,483,118 (57.99%) were multi-mapped (Supplementary Table S3). Because reads were aligned to a *de novo* transcriptome assembly rather than to a chromosome-level reference genome, the high multi-mapping rate was expected and likely reflected shared transcript sequence, isoform redundancy, paralogous transcripts, and assembly fragmentation. Therefore, downstream expression analyses were performed using gene-level abundance estimates rather than isoform-level estimates.

Transcriptome completeness was assessed using BUSCO (v5.8.2) with OrthoDB v10 as the ortholog reference. The initial run was performed using the --auto-lineage-euk flag to detect eukaryotic single-copy orthologs, followed by lineage-specific assessments using Vertebrata, Actinopterygii and Bacteroidetes databases for comparison. Required dependencies included NCBI BLAST+ (v2.16.0), HMMER (v3.4), and MetaEuk (v7.bba0d80).

To ensure removal of contaminant sequences from assembled isoforms, we iteratively ran FCS-adaptor (v0.5.5) until all the adapters were removed from the isoforms. Then, we ran FCS-GX (v0.5.5) to identify and remove foreign sequences and those sequences below 200 bp, following the NCBI Transcriptome Shotgun Assembly (TSA) submission guidelines.

### 2.9 ORF prediction

Putative coding sequences were identified using TransDecoder (v5.7.0). Long open reading frames (ORFs) exceeding 100 amino acids and exhibiting complete start/stop codons were extracted using TransDecoder.LongOrfs. ORFs were prioritized by TransDecoder.Predict. We evaluated transcript coverage of the ORFs via the Trinity script analyze_blastPlus_topHit_coverage.pl. to identify full-length candidate transcripts. CD-HIT (v4.8.1) was employed to hierarchically cluster genes with sequence similarities and remove the redundant transcripts. To ascertain the successful filtering, we ran the process twice.

### 2.10 Annotating protein-coding transcripts

To assign functional annotations, we generated homology-based annotations using UniProt protein sequences downloaded via BuildBoilerPlateTrinotate.pl. from Trinotate (v4.0.1). The reference databases, such as Uniprot and Pfam files were also retrieved from the BuildBoilerPlateTrinotate.pl. module. Homology search was conducted against both databases using BLASTp (v2.13.0+) and hmmsearch module of HMMER (v3.3.2), respectively, with E-value cut-off at 1e-05. Those genes containing “N” bases were removed. Those genes encoding proteins having poor alignment coverage (≤60%) against the Uniprot proteins were also eliminated. A total of 8,718 high-coverage BLASTp-hit Trinity gene IDs were retained after applying the 60% hit-coverage filter. These confidently annotated genes were proceeded to the downstream analysis.

### 2.11 Expression quantification

Transcript-level expression was quantified using RSEM (v1.3.3). Transcriptome references were indexed with Bowtie2 (v2.3.2) and rsem-prepare-reference. Reads from each sample were aligned using Bowtie2. Then, rsem-calculate-expression was run. Expression values were normalized across samples using the trimmed mean of M-values (TMM) method via the abundance_estimates_to_matrix.pl. script provided by Trinity, which internally utilizes edgeR (v3.42.4).

### 2.12 Processing expression values

Transcriptomic changes between tumor and matched control tissues were quantified as log₂ fold-change (log₂FC) values for each Trinity gene. Because only one tumor and one matched control sample were available, replicate-based dispersion estimation and formal differential-expression testing were not performed. Therefore, expression differences were treated as exploratory tumor-control fold change differences rather than statistically defined differential expression.

Expression values were normalized using the trimmed mean of M-values (TMM) method to correct for compositional biases and differences in sequencing depth between the two libraries. For each Trinity gene, log_2_FC was calculated from TMM-normalized expression values as:


$$\log_{2}\mathrm{FC}=\log_{2}({\mathrm{TMM}}_{\text{tumor}}+1)-\log_{2}({\mathrm{TMM}}_{\text{control}}+1)$$

Lowly expressed Trinity genes were excluded to reduce the influence of low-abundance stochastic variation. Specifically, genes with a geometric mean expression value of following equation were removed before downstream analysis:


$$\log_{2}\surd ({\mathrm{TMM}}_{\text{tumor}}+1)\;({\mathrm{TMM}}_{\text{control}}+1)\leq 1$$

Altered-expression Trinity genes were defined using a pre-defined absolute fold change threshold of ∣log₂FC∣ > 0.4, corresponding to approximately a 1.32-fold expression difference. Genes with log₂FC > 0.4 were classified as increased-expression genes, whereas genes with log₂FC < −0.4 were classified as decreased-expression genes in a tumor tissue compared to a matched control.

To evaluate the effect of threshold choice, sensitivity analyses were additionally performed using ∣log₂FC∣ > 0.3, 0.5, and 1.0. These analyses were used to assess the robustness of altered-expression gene counts and downstream GO enrichment patterns across different effect-size thresholds (Supplementary Table S4). The ∣log₂FC∣ > 0.4 threshold was selected as the primary exploratory cutoff because it provided an intermediate level of stringency, reducing the overly permissive altered-expression set while retaining sufficient genes for interpretable pathway-level analysis.

### 2.13 Functional enrichment

A total of 8,718 high coverage, BLASTp-hit genes were annotated Gene Ontology (GO) terms using extract_GO_assignments_from_Trinotate_xls.pl. from Trinotate (v4.0.1). GO enrichment analysis was conducted using the runGOSeq.pl. from Trinity (v2.14.0). GO terms associated with fewer than 50 universe genes were excluded. Only terms at hierarchy level three, as determined using the GODag function in GOATOOLS (v1.4.12), were considered. Enriched GO categories were identified using a hypergeometric test adjusted for gene length bias, with significance defined as FDR-adjusted *p*-value (i.e., q-value) < 0.05. For descriptive ranking, GeneRatio, calculated as the proportion of altered-expression genes annotated to a given term relative to the total number of genes associated with that term.

Although formal differential expression values could not be assigned to individual genes due to lack of biological replicates, GO over-representation was statistically tested conditional on the selected gene sets. Accordingly, the enrichment statistics quantify whether the altered-expression genes exhibit greater functional clustering than expected under the specified enrichment null model. They do not establish the reproducibility of the underlying gene-level expression changes across biological samples nor demonstrate generalizable biology. The GO enrichment results were therefore interpreted as exploratory and hypothesis-generating findings.

## 3 Results

### 3.1 Behavioral analysis reveals repeated contact between nasal mucosa and wall structure in captive Javanese cownose rays

**Figure 2:**
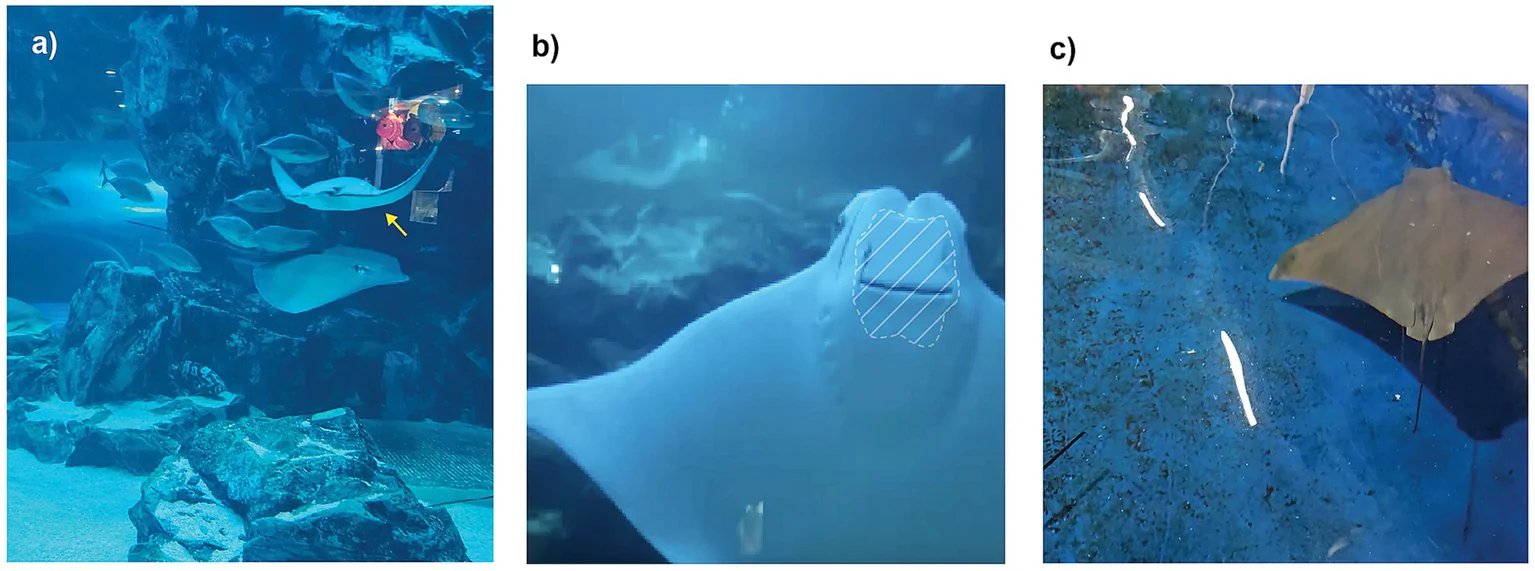
Swimming pattern of Javanese cownose rays (*Rhinoptera javanica*) under different tank conditions. **(a)** When housed in the main display tank, Javanese cownose rays predominantly swam in the midwater column (yellow arrow) or exhibited vertical climbing behavior along the tank’s acrylic wall. The main tank was consisting of floor covered with coral sand (<10 mm in grain size), and acrylic walls. **(b)** During wall-climbing behavior, the area around the mouth and nostrils (shaded area) was observed to be in consistent contact with and compressed against the acrylic wall. **(c)** When quarantined in the smaller recovery tank, the ray mostly stayed close to the bottom and swam along the floor. Note that the recovery tank floor was made of painted concrete.

Retrospective video footage of the Javanese cownose rays confirmed that, the ray habitually glided along vertical surfaces, resulting in repeated contact between the nostrils and the acrylic wall (Supplementary Video S1). More specifically, while Javanese cownose rays in the main tank were observed to swim predominantly in midwater or vertically along the acrylic walls and rarely contacting the tank floor (Figures 2a,b), those housed in the recovery tank of 1.0 m depth frequently remained close to and glided along the floor and walls (Figure 2c). While gliding, the rays’ broad anterior cephalic snout region primarily contacted the walls than other area (Figure 2b). Given that both tanks utilized identical water sources, water quality was considered a controlled variable between the two tanks. The most significant difference was the increased duration of direct contact with tank surfaces, particularly in the recovery tank. Moreover, the recovery tank featured painted concrete walls and flooring, whereas the main tank was constructed with acrylic walls and a coral sand floor (<10 mm grain size).

### 3.2 Clinical history suggests a potential link between fast tumor recurrence and housing environment

Following the first resection, the ray was housed in a recovery tank (1.0 m depth, with painted concrete walls) for easier handling during recovery. However, the tumor recurred at the same location and exhibited faster growth, resulting in a larger mass at the time of the second surgical resection performed on June 25, 2022 (4 months after the first operation) (Figure 1a). The second resection included wider margin (>0.5 cm) of surrounding tissue, which was the maximum margin achievable due to the adjacent facial structures. The ray was then housed in the recovery tank only for several days before being transferred back to the main tank. One month later, the epithelium healed well in the surgical site and the pinkish skin gradually became lighter in color. No recurrence observed after the second operation and the surgery site successfully healed.

However, the animal later developed another clinical issue (corneal opacity) and was returned to the recovery tank for regular clinical management in September 2022. The tumor recurred within a few days of transfer and subsequently exhibited rapid re-growth at the same locations in the nostrils (Figures 1b,c).

### 3.3 Histopathological findings support the fibromatous nature of the nasal tumor

**Figure 3:**
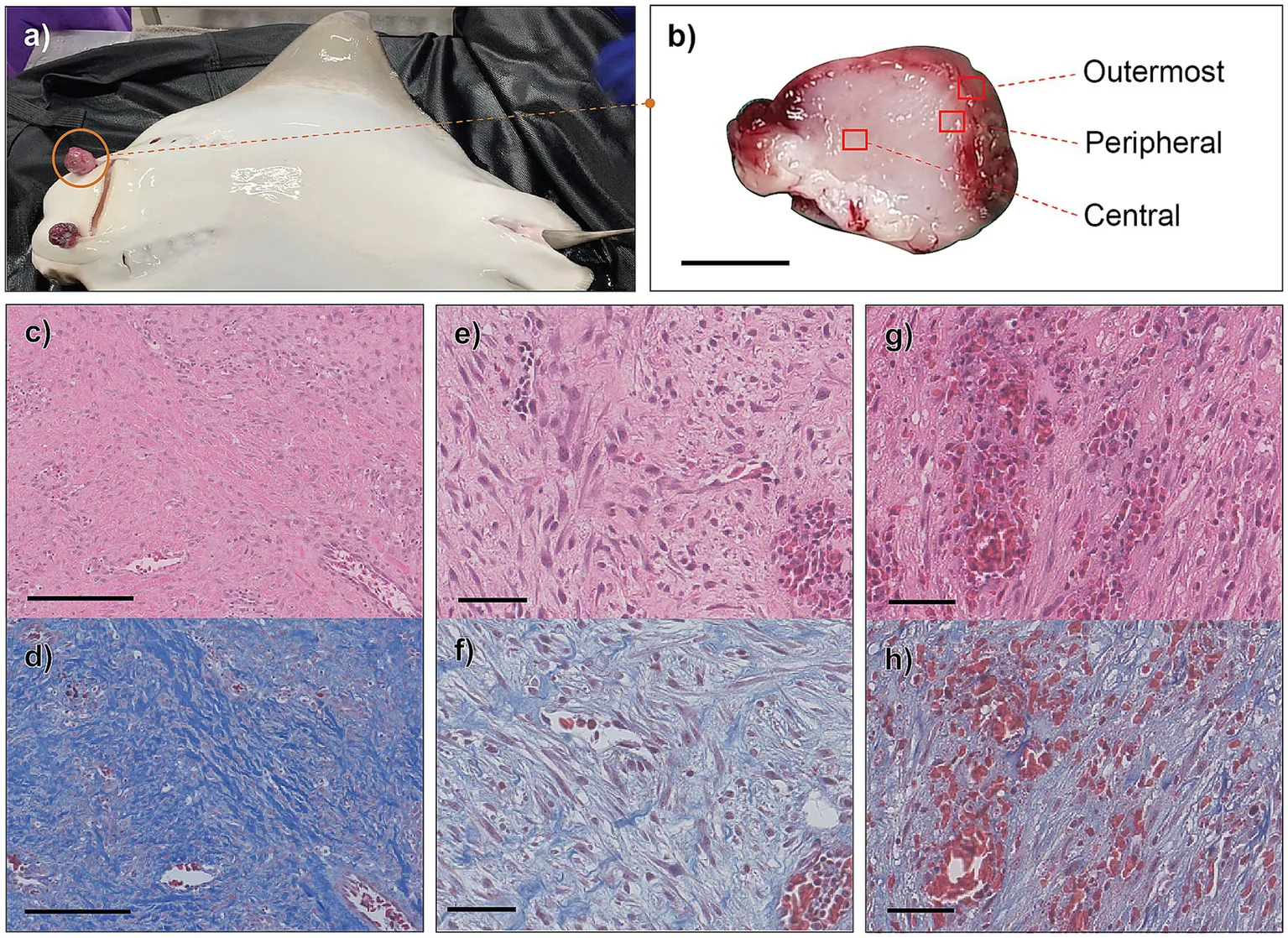
Histopathology of the Javanese cownose ray (*Rhinoptera javanica*) nasal tumor. **(a)** Anesthetized *R. javanica* on the surgery table covered with wet fabric during the second operation. Orange circle: left nasal tumor. **(b)** Cross-section of the left nasal tumor mass after resection. Red rectangles: locations of the **(c–h)**. Scale bar = 1 cm. **(c,d)** Histopathology of the central region of the tumor mass (H&E and MT stain, respectively). Dense, hypocellular fibrous stroma is filling the central region of the tumor, composed of spindle-shaped fibroblasts embedded with collagen matrix. Abundant, blue-stained collagen deposition can be found from the MT stain. Blood vessels sparsely located. Scale bars = 200 μm. **(e,f)** Histopathology of the peripheral region of the tumor mass (H&E and MT stain, respectively). Peripheral region showed increased cellularity with more prominent fibroblasts and scattered inflammatory cell aggregates, including lymphocytes and neutrophils. Collagen fibers are less densely packed with irregular orientations, showing active fibroproliferation. Scale bars = 70 μm. **(g,h)** Outermost margin of the tumor mass (H&E and MT stain, respectively). The outermost margin shows marked vascular congestion, erythrocyte extravasation, and extensive infiltration of inflammatory cells including neutrophils and mononuclear cells. The stroma was loosely organized with reduced collagen density showing focal myxoid-like stromal change. Scale bars = 50 μm.

On gross examination, the tumor masses appeared markedly congested and hemorrhagic, exhibiting a deep reddish coloration (Figure 3a). However, cross-section of the resected masses revealed hyperemia and hemorrhage confined to the outer region, which appeared reddish in color, while the central portion consisted of firm, elastic tissue with a whitish coloration (Figure 3b). To reflect this zonal architecture, histological sections were examined in a layer-specific manner.

The central region displayed features consistent with mature, scar-like fibrosis suggestive of long-standing tissue remodeling (Figure 3c). It was characterized by a hypocellular fibrous stroma with a dense and well-organized collagen matrix. Scattered spindle-shaped fibroblasts were embedded within this stroma, and minimal inflammatory infiltration was noted. Masson’s trichrome (MT) staining confirmed abundant collagen deposition, highlighted as prominent, blue-stained areas (Figure 3d). The collagen bundles were densely and concentrically arranged, indicating mature fibrosis.

In contrast, the peripheral region showed increased cellularity, with a higher density of fibroblasts and scattered aggregates of inflammatory cells, including lymphocytes and neutrophils (Figure 3e). Collagen fibers in this region were less densely packed and showed irregular orientation compared to the central region, consistent with active fibroproliferation that occurred more recently and progressed rapidly (Figure 3f). Small blood vessels were also observed, often accompanied by perivascular inflammatory infiltrates, indicating the region as an actively remodeling fibro-inflammatory zone.

The outermost region exhibited marked vascular congestion and erythrocyte extravasation, which accounted for the reddish discoloration of the tumor surface (Figure 3g). Inflammatory cells including neutrophils and mononuclear cells were abundantly present showing active inflammation. The stroma appeared pale and loosely organized, with reduced collagen density and admixed inflammatory cells, suggestive of focal myxoid-like stromal change. MT staining also demonstrated loosely arranged collagen fibers with decreased density, along with prominent inflammatory infiltrates and erythrocyte extravasation, consistent with acute injury and a reactive fibroinflammatory response (Figure 3h).

### 3.4 *De novo* transcriptome assembly of Javanese cownose ray as a genomic resource

To characterize the previously unexamined transcriptome of Javanese cownose ray, we performed deep RNA sequencing on tumor tissue and matched control samples. Combined reads from both samples were assembled *de novo*, yielding 448,972 transcripts clustered into 342,660 putative genes. The assembly spanned 282.89 Mbp with a GC content of 42.85%, an overall N50 of 1,005 bp, and a median transcript length of 322 bp (Supplementary Table S5).

To evaluate read representation in the *de novo* assembly, filtered reads were aligned to the assembled transcriptome. Of the available read pairs after quality control, 77.54% from the matched non-tumor library and 79.83% from the tumor library was mapped back to the assembly (Supplementary Table S3).

Expression-weighted contiguity analysis showed that the transcriptome was well-assembled for highly expressed genes. The Ex90N50 was 2,819 bp, encompassing 12,598 genes, indicating that the top 90% of expressed genes was reconstructed into long, contiguous sequences (Supplementary Figure S1). The initial peak at the left of the ExN50 curve is due to a small number of extremely long transcripts, such as collagen isoforms, with high expression (Supplementary Table S6).

The completeness of the transcriptome assembly was assessed using BUSCO against eukaryote, Vertebrata, and Actinopterygii lineages. Of the expected orthologs, 98.8% (252 out of 255), 85.4% (2,864 out of 3,354), and 72.8% (2,650 out of 3,640) were identified as complete, respectively (Supplementary Table S7).

Open reading frame (ORF) prediction identified 41,330 non-redundant coding sequences. Of these, 28,543 transcripts and 10,288 genes significantly matched to known eukaryotic proteins in UniProt and Pfam. Among these, 8,718 genes were classified as high coverage (alignment coverage > 60%; Supplementary Figure S2) and retained for downstream analyses (Supplementary Table S8).

### 3.5 Exploratory transcriptomic profiling identifies altered-expression patterns potentially associated with chronic mechanical stress and adaptive tissue remodeling in nasal fibroma

To explore molecular features of the nasal fibroma in Javanese cownose ray, we compared the central fibrotic core of the tumor with matched non-tumor tissue. Using the pre-defined exploratory threshold, 1,145 of 8,718 genes (13.13%) showed candidate altered expression, including 659 tumor-increased genes and 486 tumor-decreased genes (Supplementary Table S4).

**Figure 4:**
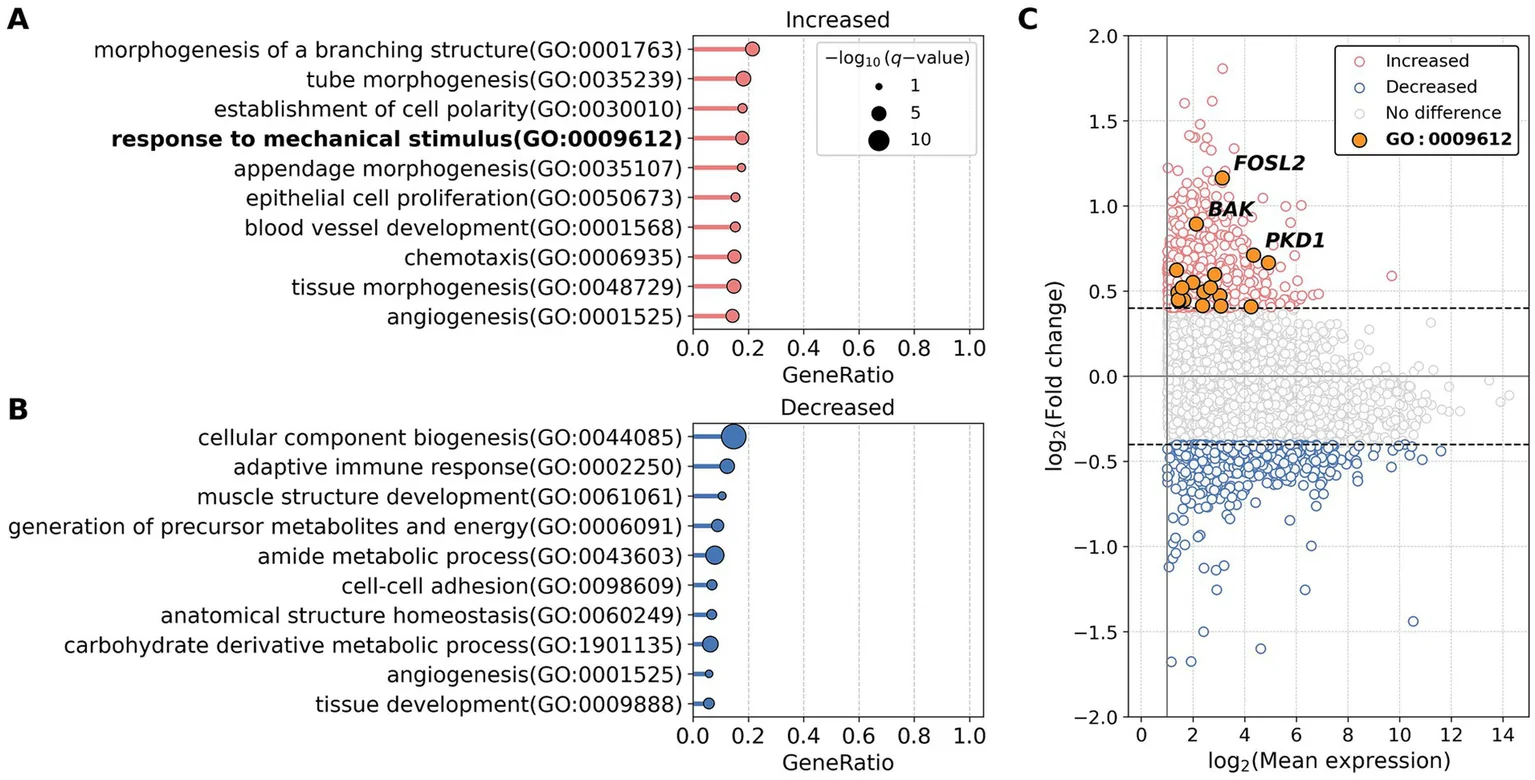
Altered-expression genes and functional enrichment associated with fibroma-specific transcriptomic changes in *Rhinoptera javanica*. **(A,B)** Lollipop plots showing the top 10 significantly enriched Gene Ontology (GO) terms for **(A)** increased genes (red) and **(B)** decreased genes (blue) in nasal fibroma tissue. The *x*-axis represents the GeneRatio (proportion of genes associated with each GO term), and the *y*-axis lists the corresponding GO terms. Dot size indicates statistical significance (i.e., −*log₁₀*(*q*-value)), with larger dots representing more significant enrichment. The GO term “response to mechanical stimulus” (GO:0009612) is highlighted in bold. **(C)** MA plot of altered gene expression between fibroma and non-tumor tissue. Each point represents a gene, colored by direction of change: Increased (red) and decreased (blue) and no difference (grey). Genes associated with GO:0009612 are highlighted in orange, with the top three genes (*FOSL2*, *BAK*, and *PKD1*) labeled. The *x*-axis shows *log₂* (mean expression), and the *y*-axis shows *log₂*(fold change). The black dashed line indicates no change in expression.

Gene ontology (GO) enrichment analysis of tumor-increased genes highlighted response to mechanical stimulus (GO:0009612) as one of the most significantly enriched terms (q = 9.29 × 10^−5^; Figure 4A, Supplementary Table S9). Key genes driving this enrichment, including FOSL2, BAK, and PKD1, exhibited pronounced increases in gene expression in tumor tissue (Figure 4C). Additional tumor-increased pathways encompassed morphogenesis of a branching structure (GO:0001763), epithelial cell proliferation (GO:0050673), and blood vessel development (GO:0001568). These patterns are consistent with a possible mechanosensitive or injury-associated tissue-remodeling response in the sampled fibromatous tumor region.

Conversely, tumor-decreased genes were predominantly involved in metabolic, biosynthetic, and immune-related processes. Enriched GO terms encompassed cellular component biogenesis (GO:0044085), adaptive immune response (GO:0002250), muscle structure development (GO:0061061), and amide metabolic processes (GO:0043603) (Figure 4B), indicative of reduced representation of cellular biosynthesis, energy-related metabolism, tissue developmental, and lymphocyte-associated activity in the fibrotic tumor tissue.

Together, altered-expression patterns in the sampled fibrotic tumor region point to increased stress-response, proliferative, and remodeling-associated activity, alongside reduced biosynthetic, metabolic, developmental, and immune-associated programs. In the context of the clinical course and histopathological findings, this transcriptomic profile provides exploratory, hypothesis-generating support for a possible mechanosensitive or injury-associated remodeling state in the fibromatous lesion.

## 4 Discussion

Clinical history gave a hint of possible correlation between housing environment and the nasal tumor occurrence. Although acrylic walls have less surface roughness than concrete wall, both still represent unnatural structures compared to the ray’s natural environment in ocean. Even minimal friction from the acrylic wall may have served as a persistent source of mechanical stimulation to the nasal mucosa, initiating pathological changes. Gliding behavior across such artificial walls therefore introduces a type of mechanical stimulus not typically experienced in the wild. Such unnatural irritation by artificial wall structures have previously been recognized in different fish species and efforts to mitigate the mechanical stress have included the use of natural substrates such as cobbles to line tank floors (16).

Interestingly, similar, albeit smaller, nasal masses have been repeatedly identified in Javanese cownose rays housed in other aquariums in the Republic of Korea. At least three additional cases were observed through visual inspection during public exhibition tours across two major facilities. These rays exhibited bilateral nasal masses that appeared nodular, congested, and occasionally hemorrhagic, closely resembling the clinical features documented in the present case. Among the diverse Chondrichthyan species housed in these aquariums, such lesions were observed exclusively in cownose rays, raising the possibility of species-specific susceptibility. However, these additional cases were identified solely on the basis of gross observations and were not confirmed by histopathological examination. Therefore, they should be interpreted cautiously and are presented only as supportive anecdotal observations.

Based on longitudinal behavioral observations by the first author (J.P.) over a six-year period and corroborating reports from aquarists at the same facility, Javanese cownose rays were consistently noted to exhibit habitual gliding along tank walls. This behavior was not observed in other ray species housed in the same environment, except occasionally during feeding. Together with this characteristic swimming behavior, this species’ uniquely broad anterior cephalic snout region resulted in frequent contact between the nasal mucosa and vertical walls. This combination of behavioral and anatomical traits likely renders the nasal mucosa of the rays vulnerable to repeated mechanical irritation. Similar cases of fibroma formation induced by chronic mechanical stress on mucosal surfaces have been well-documented in humans (17). Once a proliferative lesion develop, tumoral surface becomes more susceptible to continued mechanical stress, thereby accelerating the progression and enlargement of the tumor.

Histopathological examination confirmed the fibromatous nature of the lesion. A distinct zonal architecture was observed, with a dense collagen-rich fibrotic core and peripheral regions showing reactive inflammation, hemorrhage, vascular congestion, and stromal remodeling. These features are consistent with fibroinflammatory changes associated with sustained mechanical stress (18). Differential diagnoses included inflammatory pseudotumor, fibromatosis, and low-grade fibrosarcoma. However, the lesion formed a well-demarcated, round, firm mass composed predominantly of mature collagen-rich fibrous stroma. Inflammatory-cell infiltration was minimal within the central lesion and largely confined to the peripheral region. In addition, there was no evidence of infiltrative growth, marked cytologic atypia, or frequent mitotic activity. Collectively, these clinicopathological features were considered most consistent with fibroma. Considering the environmental conditions, clinical history, gliding behavior, and species-specific anatomical features in an integrated manner, this case of the Javanese cownose ray is consistent with a fibroma potentially associated with chronic mechanical irritation.

Analysis of altered-expression genes identified pathway-level patterns broadly consistent with the histological architecture of the sampled central fibrotic core. Tumor-increased genes were enriched for response to mechanical stimulus, epithelial cell proliferation, tissue morphogenesis, and angiogenesis, suggesting increased representation of mechanosensitive, vascular, and remodeling-associated programs in the fibromatous tissue. This aligns with the histological evidence of collagen-rich stromal remodeling and fibroinflammatory changes observed toward the peripheral tumor regions. Conversely, tumor-decreased genes were enriched for cellular component biogenesis, generation of precursor metabolites and energy, amide metabolic process, cell–cell adhesion, anatomical structure homeostasis, and adaptive immune response. These patterns are compatible with the dense, mature, relatively hypocellular character of the central fibrotic region sampled for RNA-seq, where collagen deposition was prominent and inflammatory-cell infiltration was limited. Thus, the transcriptomic profile provides an exploratory molecular view of the sampled fibrotic tumor core that complements the histological zonation of the lesion.

At the individual gene level, the expression patterns suggest interconnected mechanisms involving mechanical stimulation, immune activation, angiogenesis, extracellular matrix remodeling, and cell death in the fibroma tissue analyzed in this study. AP-1/FOSL2 drives profibrotic transcription and fibroblast differentiation (19), while mechanosensors such as PIEZO1/2 and polycystins (PKD1/2) convert chronic mechanical stimuli into Ca^2+^ dependent fibrogenic programs (20). Collagen-activated DDR2 and MMP14 orchestrate invasive extracellular matrix remodeling (21, 22). Pro-inflammatory cues (IL-1 family) interact with TGF-*β* to shape myofibroblast transitions (23), and angiogenic mediators (ANGPT2/PlGF/VEGF) help establish a fibro-vascular niche (24). KIT–SCF signaling and innate immune TBK1 pathways further potentiate fibroblast activation, aligning with the fibro-inflammatory tumor microenvironment observed in our case (25, 26). Finally, excessive activation of BAK may be associated with the induction of apoptotic pathways triggered by mechanical stress, suggesting that sustained cell death occurs under conditions of chronic inflammation and hypoxia (27).

This study has several important limitations. The transcriptomic analysis was performed on only a single tumor and matched control tissue, lacking biological and technical replication, which prevents statistical inference of differential expression. RNA-seq was performed on a bulk sample from the central fibrotic core, and therefore cannot resolve spatially distinct molecular programs across the central, peripheral, and outer regions of the lesion. No wild conspecific or independent captive control group was available, limiting the ability to distinguish species-specific, captivity-associated, and individual-level factors. Finally, because the study is observational, the proposed role of chronic mechanical irritation should be interpreted as a plausible pathogenic hypothesis rather than a demonstrated causal mechanism.

Taken together, the clinical history, tank design and material composition, behavioral observations, histopathological characteristics, and transcriptomic findings support a plausible association between repeated chronic mechanical irritation and nasal fibroma formation in this species. The three independent cases with similar gross pathological observations in other aquariums are also consistent with a possible association between suboptimal structural features of artificial captive environments and fibroma development in this species. These environmental stimuli may have contributed to transcriptional reprogramming in the sampled nasal tissue toward an inflammatory, cell proliferating phenotype, consistent with a pathological response to continuous mechanical stimulation. While this study proposes a compelling association, the absence of wild specimens for direct comparison limits the generalizability of our findings. Future research including comparative analyses with wild conspecifics will be critical to fully validate the conclusions.

## Data Availability

Raw sequencing reads are available from the NCBI Sequence Read Archive (SRA) under the BioProject accession number PRJNA1294515. RNA-Seq data of tumor tissue and matched control tissues are deposited as SRR34652969 and SRR34652970, respectively. The *de novo* transcriptome assembly generated in this study has been deposited in the NCBI GenBank database under the accession number GLHN00000000. The codes used to generate data and calculate statistics are openly available in the GitHub page: https://github.com/korean-genomics-center/FlapnoseRayTranscriptome.
