# Supplementary material for: Chronic mechanical irritation as a potential contributor to nasal fibroma formation in a captive Javanese cownose ray (Rhinoptera javanica)
Source: Front Vet Sci. 2026 Aug 12;13:1874543. doi: 10.3389/fvets.2026.1874543 (PMC13506490; doi:10.3389/fvets.2026.1874543)
Supplement: Supplementary file 1 [file Data_Sheet_1.DOCX]

**Supplementary Information**

**Chronic mechanical as a potential contributor to nasal fibroma formation in a captive Javanese cownose ray (*Rhinoptera javanica*)**

Ji-Hyung Park^1†^, Kyungwhan An^2†^, Sungwon Jeon^3^, Jong Bhak^2,3^, Seung Hyeok Seok^4,5,6,7^*, and Sang Wha Kim^1,4^*

^1^College of Veterinary Medicine & Institute of Veterinary Science, Kangwon National University, Chuncheon, Gangwon 24341, Republic of Korea

^2^Korean Genomics Center (KOGIC), Ulsan National Institute of Science and Technology (UNIST), Ulsan, 44919, Republic of Korea

^3^AgingLab, Inc., UNIST, Ulsan, 44919, Republic of Korea

^4^Macrophage Lab, Department of Microbiology and Immunology, and Institute of Endemic Disease, Seoul National University College of Medicine, Seoul 03080, Republic of Korea

^5^Department of Biomedical Sciences, Seoul National University College of Medicine, Seoul 03080, Republic of Korea

^6^Cancer Research Institute, Seoul National University, Seoul, Republic of Korea

^7^Institute of Endemic Diseases, Seoul National University Medical Research Center, Seoul, Republic of Korea

**^†^**These authors contributed equally to this work and share first authorship

** Seung Hyeok Seok (lamseok@snu.ac.kr)*

** Sang Wha Kim (sangwhakim@kangwon.ac.kr)*

| **Sample ID** | **QC Result** | **RNA sample QC** | | | | **RNA library QC** | | |
| --- | --- | --- | --- | --- | --- | --- | --- | --- |
|  |  | **Qubit (ng/µL)** | **Volume (µL)** | **Total RNA**  **(µg)** | **RIN** | **Peak Size (bp)** | **Library Concentration**  **(ng/µL)** | **Qualified yield (pmol)** |
| **Matched Normal** | Pass | 1100 | 45 | 49.5 | 8.7 | 301 | 21.4 | 3.2 |
| **Tumor Central Fibrotic Core** | Pass | 1140 | 45 | 51.3 | 8.3 | 297 | 29 | 4.4 |

**Supplementary Table 1. Summary statistics on experimental QC values during RNA library preparation.**

| **Sample ID** | **QC category** | | **Value** |  |
| --- | --- | --- | --- | --- |
|  |  |  |  |  |
| **Matched Normal** | **Raw reads before filtering** | | 210,879,874 |  |
|  | **Retained reads after filtering** | | 207,832,450 |  |
|  | **Removed reads** | **Too short** | 2,607,532 |  |
|  |  | **Excess ambiguous bases** | 430,206 |  |
|  |  | **Low quality** | 9,686 |  |
|  |  | **Total** | 3,047,424 |  |
|  | **Retention rate (%)** | | 98.55 |  |
|  | **Q30 rate before filtering (%)** | | 93.09 |  |
|  | **Q30 rate after filtering (%)** | | 94.26 |  |
|  | **Q30 improvement (%)** | | 1.1 |  |
| **Tumor Central**  **Fibrotic Core** | **Raw reads before filtering** | | 159,017,210 |  |
|  | **Retained reads after filtering** | | 156,866,896 |  |
|  | **Removed reads** | **Too short** | 1,818,064 |  |
|  |  | **Excess ambiguous bases** | 326,188 |  |
|  |  | **Low quality** | 6,062 |  |
|  |  | **Total** | 2,150,314 |  |
|  | **Retention rate (%)** | | 98.65 |  |
|  | **Q30 rate before filtering (%)** | | 93.13 |  |
|  | **Q30 rate after filtering (%)** | | 94.22 |  |
|  | **Q30 improvement (%)** | | 1.09 |  |

**Supplementary Table 2. Read preprocessing and summary quality statistics for RNA-seq libraries.** Raw paired-end RNA-seq reads from matched normal tissue and the tumor central fibrotic core were processed using fastp. Raw reads before filtering indicate the total number of sequencing reads generated before preprocessing. Retained reads after filtering represent reads that passed adapter trimming, fixed-end trimming, quality filtering, and ambiguous-base filtering. Removed reads are subdivided by the primary filtering reason: “too short” indicates reads discarded after trimming because their remaining length was insufficient; “excess ambiguous bases” indicates reads containing more ambiguous nucleotides than allowed; and “low quality” indicates reads failing the minimum average quality requirement. The retention rate represents the percentage of raw reads retained after preprocessing. Q30 rates before and after filtering indicate the percentage of bases with Phred quality scores of 30 or higher before and after preprocessing, respectively, and Q30 improvement indicates the absolute increase in Q30 rate after filtering.

| **Sample ID** | **Mapping category** | **Read-pair count** | **Proportion of  mapped pairs (%)** |
| --- | --- | --- | --- |
| **Matched Normal** | Retained read pairs | 103,916,225 | - |
|  | Total mapped pairs | 80,572,107 | 77.54 |
|  | Uniquely mapped pairs | 21,528,256 | 20.72 |
|  | Multi-mapped pairs | 59,043,851 | 56.82 |
| **Tumor Central Fibrotic Core** | Retained read pairs | 78,433,448 | - |
|  | Total mapped pairs | 62,613,361 | 79.83 |
|  | Uniquely mapped pairs | 17,130,243 | 21.84 |
|  | Multi-mapped pairs | 45,483,118 | 57.99 |

**Supplementary Table 3. Read-pair mapping statistics for RNA-seq libraries aligned to the *de novo* *Rhinoptera javanica* transcriptome assembly.** Trimmed paired-end reads from the matched normal tissue and the tumor central fibrotic core were aligned back to the assembled transcriptome. Retained read pairs show the total number of read pairs after quality filter. Proportion of mapped pairs of the retained read pairs are indicated by “-” as it is the original number of read pairs before aligning. Total mapped pairs indicate all read pairs aligned to the transcriptome. Uniquely mapped pairs represent read pairs with a single best alignment, whereas multi-mapped pairs represent read pairs with multiple transcript-level alignments. Percentages were calculated relative to the retained read pairs within each sample.

| **absolute log_2_  fold-change  threshold** | **Number of altered- expression genes** | | | **Number of genes  in universe** | **Proportion of altered genes (%)** |
| --- | --- | --- | --- | --- | --- |
|  | **increased** | **decreased** | **total** |  |  |
| **0.3** | 1,031 | 1,166 | 2,197 | 8,718 | 25.2 |
| **0.4** | 659 | 486 | 1,145 | 8,718 | 13.13 |
| **0.5** | 432 | 196 | 628 | 8,718 | 7.2 |
| **1** | 41 | 14 | 55 | 8,718 | 0.63 |

**Supplementary Table 4.** **Sensitivity analysis of altered-expression gene counts across absolute log₂ fold-change thresholds**. Trinity genes with altered expression were defined using progressively stringent absolute log₂ fold change thresholds. Increased-expression genes were defined as genes with log_2_FC greater than the indicated threshold, whereas decreased-expression genes were defined as genes with log_2_FC less than the negative of the indicated threshold. The universe comprised 8,718 expressed high-confidence BLASTp-hit Trinity genes used as the background for downstream GO enrichment analysis.

| Counts of transcripts | |
| --- | --- |
| Total Trinity genes  (*pre-filtering) | 352,660  (373,778) |
| Total Trinity transcripts  (*pre-filtering) | 448,972  (473,086) |
| GC (%) | 42.85 |
| Statistics based on ALL transcript contigs | |
| Contig N50 (bp) | 1,005 |
| Median contig length (bp) | 322 |
| Average contig length (bp) | 630.08 |
| Total assembled bases (bp) | 282,888,771 |
| Statistics based on ONLY LONGEST ISOFORM per ‘GENE’ | |
| Contig N50 (bp) | 568 |
| Median contig length (bp) | 306 |
| Average contig length (bp) | 507.53 |
| Total assembled bases (bp) | 178,985,589 |

**Supplementary Table 5. Summary statistics of the de novo transcriptome assembly for *Rhinoptera javanica*.** Assembly metrics are shown for all transcript contigs and for the longest isoform per Trinity-defined gene. Metrics include the total number of genes and transcripts, GC content, contig N50, median and average contig length, and total assembled base. *pre-filtering = before removing foreign and barcode contaminations detected by FCS.

| Transcript ID | Protein name | Length(bp) | Mean expression | Cumulative expression percentage (%) |
| --- | --- | --- | --- | --- |
| TRINITY_DN1318_c0_g1_i2 | Collagen alpha-1(I) chain | 5147 | 28957.8 | 3.3 |
| TRINITY_DN259_c9_g1_i2 | Collagen alpha-1(I) chain | 1357 | 16854.4 | 5.3 |
| TRINITY_DN199_c8_g2_i1 | Unknown | 1131 | 15659.5 | 7.1 |
| TRINITY_DN199_c153_g1_i1 | Unknown | 567 | 14693.9 | 8.8 |
| TRINITY_DN207455_c9_g1_i1 | Unknown | 2025 | 14647.2 | 10.5 |
| TRINITY_DN282_c1_g1_i4 | Unknown | 867 | 14620.6 | 12.2 |
| TRINITY_DN142_c49_g1_i3 | Fibronectin | 777 | 10043.9 | 13.3 |
| TRINITY_DN429_c2_g2_i3 | SPARC | 2421 | 9717.2 | 14.4 |
| TRINITY_DN3817_c12_g1_i1 | Unknown | 1690 | 9500.2 | 15.5 |
| TRINITY_DN10_c0_g1_i2 | Collagen alpha-2(I) chain | 5762 | 9309.2 | 16.6 |
| TRINITY_DN5119_c1_g1_i1 | Unknown | 641 | 6416.7 | 17.3 |
| TRINITY_DN10_c0_g1_i4 | Collagen alpha-2(I) chain | 5762 | 5372.9 | 18 |
| TRINITY_DN36_c0_g2_i1 | Unknown | 2512 | 5207.1 | 18.6 |
| TRINITY_DN176931_c5_g1_i1 | Ferritin heavy chain | 1158 | 5182.3 | 19.2 |
| TRINITY_DN50_c0_g3_i2 | Unknown | 529 | 5084.9 | 19.8 |

**Supplementary Table 6. Top expressed transcripts in the *Rhinoptera javanica* nasal tissue transcriptome**. The table lists the highest expressed transcripts ranked by mean expression (TMM-normalized counts). For each transcript, the transcript identifier, putative protein annotation, transcript length (bp), arithmetic mean expression, and cumulative expression percentage of total transcriptome expression are provided. Collagen isoforms (alpha-1 and alpha-2 chains) are among the most abundantly expressed transcripts, reflecting the fibrous extracellular matrix composition of nasal tissue, while other highly expressed transcripts include structural and stress-response proteins. Cumulative expression percentage indicates the proportion of total transcriptome expression accounted for by each transcript.

| Categories | | eukaryote | *vertebrata* | *actinopterygii* | *bacteroidetes* |
| --- | --- | --- | --- | --- | --- |
| Total BUSCO groups | | 255 | 3,354 | 3,640 | 402 |
| Complete BUSCOs | all  (C=S+D) | 252 (98.8%) | 2,864 (85.4%) | 2,650 (72.8%) | 33 (8.2%) |
|  | single-copy (S) | 124 (48.6%) | 1,368 (40.8%) | 1,288 (35.4%) | 15 (3.7%) |
|  | duplicated  (D) | 128 (50.2%) | 1,496 (44.6%) | 1,362 (37.4%) | 18 (4.5%) |
| Fragmented | | 2 (0.8%) | 135 (4.0%) | 64 (1.8%) | 3 (0.7%) |
| Missing | | 1 (0.4%) | 355 (10.6%) | 926 (25.4%) | 366 (91.0%) |

**Supplementary Table 7. BUSCO assessment of transcriptome completeness for *Rhinoptera javanica*.** The evaluation was performed against three lineage-specific ortholog databases: eukaryote (n = 255), *vertebrata* (n = 3,354), *actinopterygii* (n = 3,640) for positive controls and *bacteroidetes* (n = 402) as a negative control. Categories reported include the number and percentage of complete BUSCOs (C or all), further sub-divided into single-copy (S) and duplicated (D), as well as fragmented and missing BUSCOs.

| Categories | Number of ORFs |
| --- | --- |
| Longest ORFs | 81,062 |
| Predicted ORFs | 41,403 |
| Non-redundant ORFs | 41,330 |
| BLASTp-hit Isoforms | 28,543 |
| BLASTp-hit Genes | 10,288 |
| High coverage, BLASTp-hit Isoforms | 17,391 |
| High coverage, BLASTp-hit Genes | 8,718 |

**Supplementary Table 8. Summary statistics of open reading frame (ORF) prediction and gene filtering in the *Rhinoptera javanica* transcriptome.** The table reports the number of ORFs and genes based on coding potential, ORF length, and homology evidence.

| **Category** | **GO BP Term (GO ID)** | **Input** | **Univ** | **Gene Ratio** | **q** | **-log_10_(q)** |
| --- | --- | --- | --- | --- | --- | --- |
| **tumor- increased** | morphogenesis of a branching structure (GO:0001763) | 17 | 79 | 0.22 | 2.85E-05 | 4.55 |
|  | tube morphogenesis (GO:0035239) | 23 | 126 | 0.18 | 8.71E-06 | 5.06 |
|  | establishment of cell polarity (GO:0030010) | 12 | 67 | 0.18 | 1.01E-02 | 2.00 |
|  | response to mechanical stimulus (GO:0009612) | 18 | 101 | 0.18 | 9.29E-05 | 4.03 |
|  | appendage morphogenesis (GO:0035107) | 10 | 57 | 0.18 | 2.85E-02 | 1.55 |
|  | epithelial cell proliferation (GO:0050673) | 14 | 91 | 0.15 | 1.41E-02 | 1.85 |
|  | blood vessel development (GO:0001568) | 17 | 111 | 0.15 | 4.91E-03 | 2.31 |
|  | chemotaxis (GO:0006935) | 22 | 147 | 0.15 | 8.05E-05 | 4.09 |
|  | tissue morphogenesis (GO:0048729) | 30 | 203 | 0.15 | 2.37E-05 | 4.62 |
| **tumor- decreased** | cellular component biogenesis (GO:0044085) | 28 | 190 | 0.15 | 3.97E-15 | 14.40 |
|  | adaptive immune response (GO:0002250) | 13 | 105 | 0.12 | 1.02E-05 | 4.99 |
|  | muscle structure development (GO:0061061) | 6 | 57 | 0.11 | 3.15E-02 | 1.50 |
|  | generation of precursor metabolites and energy (GO:0006091) | 13 | 146 | 0.09 | 2.97E-04 | 3.53 |
|  | amide metabolic process (GO:0043603) | 28 | 354 | 0.08 | 1.56E-08 | 7.81 |
|  | cell-cell adhesion (GO:0098609) | 14 | 204 | 0.07 | 3.87E-03 | 2.41 |
|  | anatomical structure homeostasis (GO:0060249) | 13 | 192 | 0.07 | 5.34E-03 | 2.27 |
|  | carbohydrate derivative metabolic process (GO:1901135) | 31 | 494 | 0.06 | 1.49E-06 | 5.83 |
|  | angiogenesis (GO:0001525) | 11 | 189 | 0.06 | 4.77E-02 | 1.32 |
|  | tissue development (GO:0009888) | 20 | 348 | 0.06 | 1.79E-03 | 2.75 |

**Supplementary Table 9. Summary statistics of Gene Ontology (GO) analysis on candidate altered-expression genes in the nasal fibroma.** Top 10 ranking GO terms for tumor-increased and tumor-decreased gene sets are shown. “Category” indicates whether the GO term was enriched among increased- or decreased-expression genes. “GO BP Term” provides the enriched Gene Ontology: Biological Process term and its corresponding GO identifier. “Input” denotes the number of altered-expression genes from the analyzed gene set that were annotated to the indicated GO term. “Univ” denotes the total number of genes annotated to that GO term in the background gene universe. “GeneRatio” represents the proportion of GO-annotated background genes captured in the input set, calculated as Input divided by Univ. “q” indicates the FDR-adjusted enrichment p-value, with smaller values reflecting stronger statistical support after multiple-testing correction. “-log10(q)” is the negative base-10 logarithm of the q-value, used to display enrichment strength on a positive scale; larger values correspond to smaller q-values and stronger enrichment support.

**Supplementary Figure 1. Expression-weighted contig N50 (ExN50) of the *Rhinoptera javanica* transcriptome.** The ExN50 metric integrates transcript abundance into contiguity assessment by plotting contig N50 length (y-axis) against the cumulative proportion of normalized expression (Ex, x-axis). The highlighted point marks the Ex90N50 value of 2,819 bp, derived from 12,598 genes, indicating the contig length at which 90% of the total normalized expression is captured.

**Supplementary Figure 2. Alignment coverage distribution of predicted protein-coding transcripts based on BLASTp alignment.** The x-axis represents the percentage of each query sequence aligned (alignment coverage, %), and the y-axis indicates the number of transcripts in each coverage bin. The red dashed line denotes the 60% hit coverage threshold to define confident transcript matches during functional annotation.

**Supplementary Video 1. Characteristic swimming behavior of the Javanese cownose ray (*Rhinoptera javanica*).** Representative footage of a healthy Javanese cownose ray exhibiting its characteristic swimming pattern under normal conditions. Unlike other cohabiting species, which rarely swim along vertical surfaces, the ray frequently glides upward along the aquarium’s acrylic walls. During this wall-climbing behavior, the broad anterior cephalic region repeatedly makes contact with the wall surface. This species-specific pattern highlights the tendency of cownose rays to engage in wall-gliding locomotion, which may result in repetitive mechanical contact between the nasal mucosa and tank structures.
